# Two-Stream Bidirectional Interaction Network Based on RGB-D Images for Duck Weight Estimation

**DOI:** 10.3390/ani15071062

**Published:** 2025-04-06

**Authors:** Diqi Zhu, Shan Bian, Xiaofeng Xie, Chuntao Wang, Deqin Xiao

**Affiliations:** 1 College of Mathematics and Informatics, South China Agricultural University, Guangzhou 510642, China; 2 Key Laboratory of Smart Agricultural Technology in Tropical South China, Ministry of Agriculture and Rural Affairs, Guangzhou 510642, China

**Keywords:** duck weight estimation, cross-modality, RGB-D dataset

## Abstract

An automated non-contact weight measurement method for ducks is beneficial for preventing the stress response of ducks and, thus, promoting their healthy development. We propose a two-stream bidirectional interaction network that depends on RGB-D pictures to accurately determine the weight of ducks. We developed two-stream branches in the encoder to extract texture appearance information and spatial structure information from RGB images and depth images, respectively. Besides, we employed a cross-modality feature supplement module in the encoder to facilitate mutual learning and complementarity between these two modalities. Finally, a decoder is designed to combine the multi-scale characteristics of these two modalities and feed the fused features into the regression module to determine the final weight of the duck. For the experimental analysis of this study, we built a new dataset of RGB-D duck images consisting of 2865 pairs of RGB-D images captured from the bird-eye view. The comparative experimental results show that the proposed method could effectively estimate the duck weight with an MAE of only 0.1550, outperforming all the comparison methods on this dataset. This automated, non-contact weight measurement method can eliminate stress responses caused by human intervention. This method enables the automated collection of growth data, supporting precision feeding and health management decisions. It drives the digital and welfare-oriented transformation of the livestock industry, enhancing production efficiency while promoting animal welfare and sustainable agricultural practices.

## Simple Summary

Non-contact weight measurement in ducks is critical for reducing duck stress and enabling precision poultry management. We propose an automated method using RGB-D images and a two-stream bidirectional network to estimate duck weight accurately. The method leverages separate branches in the encoder to extract RGB and depth features, complemented by cross-modal fusion to enhance feature complementation between modalities. The decoder then combines multi-scale features for weight regression. Validated on a novel dataset of 2865 RGB-D duck images, our approach achieves the lowest MAE of 0.1550, outperforming existing methods. This technology eliminates physical handling stress, automates growth data collection, and supports informed decisions for precision feeding and health monitoring. By promoting animal welfare, it advances sustainable agricultural practices in the poultry industry.

## 1. Introduction

China has maintained its leadership role in the duck breeding industry, with its duck population exceeding 60% of the world’s ducks in 2019 [1]. Duck farms can benefit from timely information regarding individual ducks to analyze duck development and increase duck meat production. Duck body weight, being a significant body parameter, can assist poultry farmers in regulating feed quantity and assessing the health of their flocks. Initially, ducks were typically assessed using the direct weighing method, which involved placing the ducks on a scale for determination. An alternative approach employed the manual assessment of duck body measurements to estimate body weight, relying on the correlation between body weight and body measurements. Téguia et al. (2008) [2] investigated the precision with which dimension characteristics could be utilized for estimating the body weights of ducks. Nevertheless, the implementation of these techniques incurs high labor costs and may induce stress in ducks, reducing their productivity.

The development of computer vision has brought great interest in the field of agriculture, particularly in image-based weight estimation algorithms. For large livestock, such as pigs, cows and sheep, the complexity of body size and physiological characteristics leads to difficulties in weight estimation. As for small poultry, their rapid movement, group housing and shading lead to difficulties in weight estimation. Li et al. (2020) [3] proposed an automated monitoring method based on image processing and machine learning, which achieved the precise recognition of feeding and drinking behaviors in group-reared broilers. Xiao et al. (2024) [4] developed the DHSW-YOLO model using deep learning, optimizing the YOLOv8 network architecture to enable real-time recognition of duck flock behaviors under both bright and dark conditions. These studies provide low-cost and highly robust behavioral monitoring tools for smart farming, laying a technical foundation for optimizing resource allocation, improving animal welfare, and enhancing production efficiency. With the aid of image processing techniques, the weights of ducks can be accurately estimated without human contact. This approach not only minimizes manual intervention to prevent stress on the ducks but also helps in reducing labor costs. A typical approach in previous studies is placing a camera in an elevated position to take pictures of ducks and then analyzing the images to determine their weight by extracting relevant features. The conventional approach involves analyzing the RGB images and determining the weight through regression models that utilize human-determined characteristics. Ogah et al. (2011) [5] studied the relationship between body weight and various physical size characteristics of male and female ducks. Via factor analysis and multiple regression, the authors determined that these characteristics had a statistically significant positive impact. Besides, Lieng and Sangpradit (2020) [6] successfully estimated the body weight of ducks by employing image processing techniques on top-view images of ducks and extracting their characteristic body features. Furthermore, Chen et al. (2023) [7] employed a Convolutional Neural Network (CNN) to predict the weight of duck carcasses. The CNN-based method demonstrated superior precision and accuracy compared to the traditional regression model. However, relying solely on RGB features may result in the loss of spatial structure information, which is crucial for enhancing the accuracy of duck weight estimation.

Due to the advances in 3D photography technology, RGB-D cameras have increased in popularity for capturing depth information of animals. Pezzuolo et al. (2018) [8] used Microsoft Kinect to collect depth images to extract the physical dimensions of a pig’s body and subsequently estimate its weight using a nonlinear model. Deep learning has become increasingly utilized in various image-processing applications in recent years. Cang et al. (2019) [9] proposed a pig weight estimation model that employs Faster R-CNN. This model uses a top-view depth image of a pig as input and is capable of simultaneously performing pig identification, localization, and body weight estimation. He et al. (2021) [10] developed a network with a two-branch BotNet Antonakakis et al. (2017) [11] and depth images as input for pig weight estimation, with an MAE of 6.366. Depth images contain significant structural information in comparison to RGB images. Nevertheless, the sole depth modality does not possess the same level of texture appearance information as the RGB modality. To address the problems mentioned above, the researchers focus on the task of estimating animal weight by utilizing both RGB images and depth images, thereby maximizing the unique benefits of each modality. He et al. (2023a) [12] proposed an algorithm for estimating the weight of sheep using a model based on LiteHRNet. The algorithm takes a four-channel image that combines RGB images and depth images as input. The algorithm achieves a mean percentage error (MAPE) of 14.605%. He et al. (2023b) [13] proposed a two-stream cross-attention Transformer network based on RGB and depth images, which learns feature information from RGB and depth images separately for the task of estimating the weight of the pig, and its MAE is 3.237.

To the best of our knowledge, there is currently a lack of research utilizing RGB-D data for duck weight estimation. To address this, we have developed an algorithm that estimates duck weight using a two-stream bidirectional interaction network based on RGB-D images. The algorithm comprises an encoder, a decoder and a regression module to extract features from both modalities fully. The encoder extracts features from both RGB and depth images using the MobileNet backbone and incorporates bidirectional interactions between the two modalities to improve communication. The decoder contains a feature aggregation operation combining multi-modality multi-scale features to produce the ultimate fusion feature. In the end, a regression module was developed for precisely determining the duck’s weight. We present the design of an image acquisition system for conducting experiments and obtaining a new dataset of RGB-D images of ducks. Subsequently, we perform ablation experiments and comparison experiments to validate the efficacy of the proposed method, which achieves better results on the test dataset, yielding an MAE of 0.1550.

The main contributions of this paper can be summarized as follows:

- We construct an RGB-D duck weight dataset named SCAU-DuckWT to aid in the research of duck weight estimation. The dataset includes RGB-D images of ducks captured from the bird-eye perspective and the corresponding weight data.
- We present a two-stream RGB-D network for estimating duck weights that can utilize the spatial information of depth images and the texture appearance information of RGB simultaneously.
- We utilize an encoder–decoder architecture with a bidirectional interaction module in the encoder. This allows us to learn a shared representation by capturing the interactions between RGB and depth images. Next, the decoder combines the multi-scale features from both modalities.
- The proposed method provides the best performance for duck estimation when compared to other methods, with an MAE of only 0.1550.

## 2. Materials and Methods

The following section introduces our newly obtained SCAU-DuckWT dataset, followed by an elaborate explanation of the proposed approach and a description of the experimental setup.

### 2.1. Dataset

**Figure 1:**
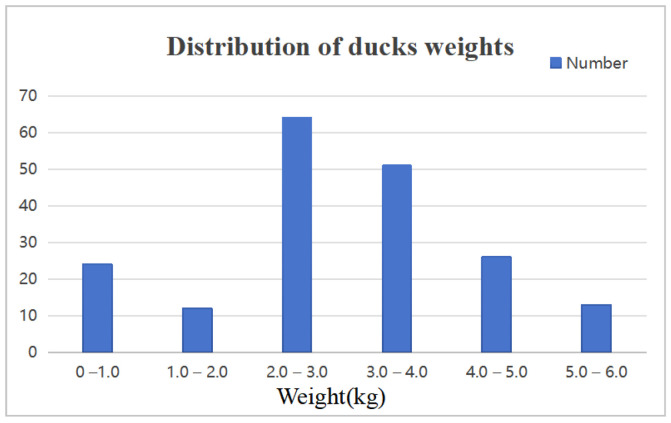
The weight distribution of ducks.

The SCAU-DuckWT dataset includes pairs of RGB images and depth images. At present, the dataset utilized for duck weight estimation comprises a solitary RGB or depth image, which is insufficient to train the model proposed in this paper. To address the issue, we developed an image acquisition system specifically designed to gather a novel RGB-D dataset for training. The data were acquired from Zengcheng Teaching and Research Bases, South China Agricultural University, located in Guangdong Province, China. In our current experimental environment, ducks are reared in cages. The confined nature of the cage environment limits the ducks’ movement, resulting in relatively fewer dynamic behaviors, such as frequent walking, wing flapping, or sudden bursts of activity. We utilized a group size of 200 ducklings as our study subjects and collected data throughout the entire duckling period. Every week, a total of 24 ducks were randomly selected for image collection and weight recording. This process continued for a period of eight weeks. The ultimate body weights varied from 0.50 kg to 5.50 kg. Figure 1 displays the weight distribution of the final dataset.

**Figure 2:**
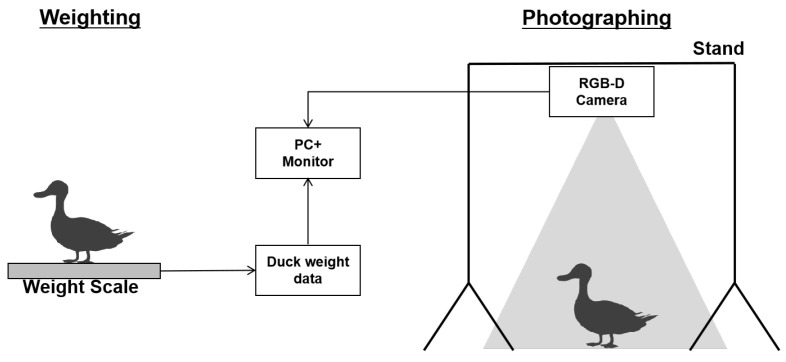
The infrastructure of equipment.

The image collecting and weight recording of ducks is a challenging task. We divided the process into two separate steps to ensure precise weight measurements and high-quality image capture. The chosen ducks were first weighed and then directed to the designated area for capturing images. We established a shooting area with dimensions of 475 mm by 680 mm and positioned a camera at a height of 100 cm above to capture an aerial view. The depth camera used is an Obi-Zoom Depth Camera linked to a laptop computer to capture image data. The Obi-Zoom Depth Camera can capture both color and depth information simultaneously. It offers an RGB resolution of 1920 × 1080 pixels and a depth resolution of 640 × 480 pixels, and its frame rate is 30 FPS. The depth measurement range of this camera spans from 0.5 m to 5 m. Figure 2 displays the complete structure of the experimental setup.

**Figure 3:**
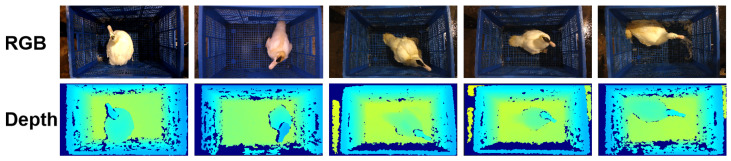
The RGB-D images of raw samples. The above are RGB images, and the below are depth images.

After acquiring the duck images, we manually filtered these images to eliminate those blurry or lacking spatial information and then paired the duck images with weight data. In the data pre-processing stage, we applied Gaussian filtering to smooth the images and carried out color normalization processing on the RGB images to ensure color consistency among different images. Meanwhile, online data augmentation operations were applied, including random aspect ratio cropping, random cropping, random horizontal flipping, random vertical flipping, random rotation, color jittering, and Gaussian blur. Ultimately, we ended up with 2865 pairs of RGB-D images to form the SCAU-DuckWT dataset. Among these, 2325 pairs were randomly selected for training, while the remaining 540 pairs were designated for testing. The illustration of the dataset containing ducks is depicted in Figure 3.

### 2.2. Methods

Based on the SCAU-DuckWT dataset, we designed a two-stream bidirectional interaction network that utilizes both RGB and depth modalities to accurately determine the weight of ducks. To effectively utilize the characteristics of both modalities, we adopted the idea of RGB-D based SOD (Salient Object Detection) in the design of our model. Salient object detection is an approach that emulates the human visual perception system to identify the most visually appealing targets in a scene (Zhou et al., 2021) [14]. In RGB-D based SOD models, the crucial step is to efficiently extract and combine the feature from both the RGB image and the depth image. Most models achieve this by using two-stream networks. A two-stream network is composed of two independent branches that individually process RGB images and depth images to produce distinct high-level features. These features are subsequently combined using various fusion techniques. The early fusion approach involves inputting RGB and depth images into separate networks and merging their low-level representations into a unified representation. This combined representation is then passed into a subsequent network for further processing (Qu et al., 2017) [15]. Later fusion employs two parallel neural networks to independently learn the high-level characteristics of RGB and depth images and then combines them to produce the ultimate high-level features (Han et al., 2017) [16]. The multi-scale fusion technique combines RGB images and depth images in a multi-scale multi-path fusion network. Additionally, a cross-modal interaction module is incorporated to improve the complementarity of various features (Chen et al., 2019) [17]. This section provides a concise overview of the network framework and then presents the key components of the method, i.e., the feature extraction encoder and the cross-modal feature fusion decoder, etc.

#### 2.2.1. Overview of Proposed Method

**Figure 4:**
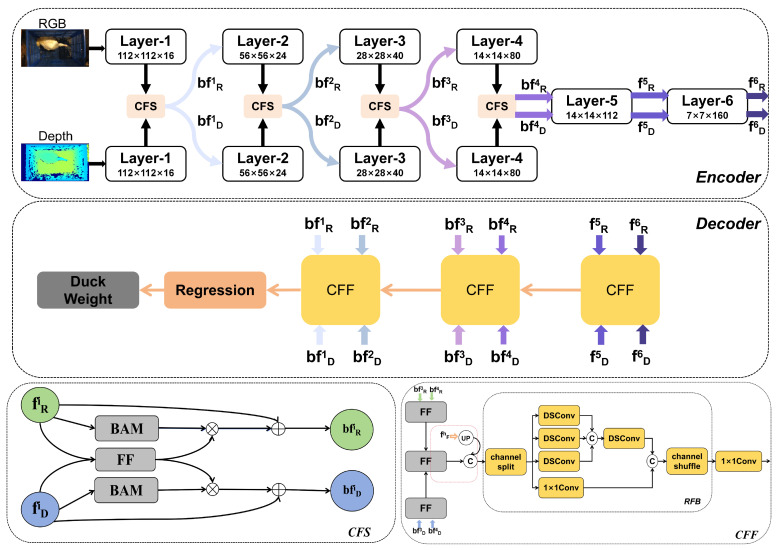
The architecture of the proposed network.

Figure 4 illustrates our proposed framework for the duck weight estimation network. The network is based on the TBINet network (Wang and Zhang, 2022) [18] for RGB-D SOD, composed of an encoder, decoder and regression module. The encoder component utilizes the MobileNet-v3 large architecture (Howard et al., 2019) [19] consisting of both the RGB branch and depth branch. These two branches employ a bidirectional communication strategy to produce features that are associated with the weight of ducks. The primary function of the encoder is to derive the feature representation of the input image. The decoder component aggregates the features obtained from the encoder and produces the ultimate feature map. The objective of the decoder is to progressively fuse and up-sample the low-level features extracted by the encoder to generate high-level semantically rich features. Ultimately, these characteristics are input into the regression module that uses the combined features from the decoder to estimate the weight of the duck. Specifically, the encoder was partitioned into six layers, following the structure of the MobileNet-v3 large network. The output features of the *i*-th layer in both the RGB branch and the depth branch are denoted as $f_{M}^{i}\left(M\in \left\{R,D\right\},i=1,\ldots ,6\right)$. In the initial four layers, we employed the cross-modality feature supplementation (CFS) module and the resulting features from the CFS module are represented as $bf_{M}^{i}\left(M\in \left\{R,D\right\},i=1,\ldots ,4\right)$. Once the encoding process is complete, the decoder receives both $bf_{M}^{i}\left(M\in \left\{R,D\right\},i=1,\ldots ,4\right)$ and $bf_{M}^{i}\left(M\in \left\{R,D\right\},i=5,6\right)$ as input. The decoder is comprised of three Cross-Modality Feature Fusion (CFF) modules. The fused features are finally input into the regression module to derive the final weight outcome.

The following sections provide a detailed introduction to the encoder module for multi-modality feature extraction and the decoder module for cross-modality feature fusion in the network.

#### 2.2.2. Cross-Modal Feature Extraction Encoder

**Figure 5:**
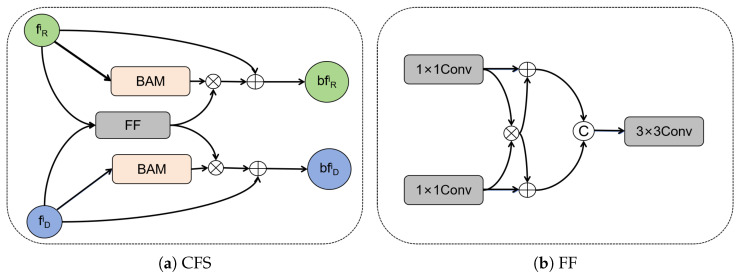
Illustration of the proposed cross-modality feature supplement (CFS) module and feature fusion (FF) module.

The encoder of the proposed model adopts the two-branch network with six layers for extracting various scale features, as illustrated in Figure 4. Within the initial four layers, we utilized cross-modality feature supplementation within each layer. These complemented features are subsequently passed on to the next layer, effectively utilizing cross-modality information to enhance the feature extraction. To fuse these two modalities fully, we propose a module called Cross-Modality Feature Supplementation (CFS) as shown in Figure 5a. The CFS module incorporates a feature fusion (FF) module, which efficiently and straightforwardly fuses cross-modality features as depicted in Figure 5b.

Given features $f_{R}^{i}\left(i=1,\ldots ,4\right)$ and $f_{D}^{i}\left(i=1,\ldots ,4\right)$, they are initially passed through a 1×1 convolutional layer to modify their channel counts and obtain their smoothed feature representations ${\bar{f}}_{R}^{i}=Conv_{1\times 1}\left(f_{R}^{i}\right)$ and ${\bar{f}}_{D}^{i}=Conv_{1\times 1}\left(f_{D}^{i}\right)$. Next, we employ element-wise multiplication to highlight the common feature representations ${\bar{F}}_{f}^{i}={\bar{f}}_{R}^{i}⊗{\bar{f}}_{D}^{i}$, with ⊗ representing element-wise multiplication. The enhanced features are combined and input into the depth-separable convolutional layer to obtain the final fused features. The process can be described as follows:

(1) $$\begin{array}{c} \begin{array}{c} f_{F}^{i}=DSConv_{3\times 3}\left(\left[{\bar{f}}_{F}^{i}+{\bar{f}}_{R}^{i},{\bar{f}}_{F}^{i}+{\bar{f}}_{D}^{i}\right]\right) \end{array} \end{array}$$

After we acquired the fused feature $f_{F}^{i}$, we utilized the BAM (Bottleneck Attention Module) (Park et al., 2018) [20] attention mechanism to enhance the $f_{F}^{i}$. Subsequently, we merged the enhanced features with the original features from both modalities. The BAM attention mechanism is a constraining attention module that can be incorporated into any feed-forward convolutional neural network to deduce attention mapping through both the channel and spatial paths. The process is as stated in Equations (2) and (3).

(2) $$\begin{array}{c} \begin{array}{c} bf_{R}^{i}=f_{R}^{i}+\sigma \left(BAM\left(f_{R}^{i}\right)\right)⊗f_{F}^{i} \end{array} \end{array}$$

(3) $$\begin{array}{c} \begin{array}{c} bf_{D}^{i}=f_{D}^{i}+\sigma \left(BAM\left(f_{D}^{i}\right)\right)⊗f_{F}^{i} \end{array} \end{array}$$

After the CFS modules, the features $bf_{R}^{i}\left(i=1,\ldots ,4\right)$ and $bf_{D}^{i}\left(i=1,\ldots ,4\right)$ are input into the subsequent layer of the encoder as well as the decoder. Finally, these improved fusion features are transferred to the two modality branches in a complementary manner. The CFS module efficiently eliminates the low-quality depth information and transmits high-quality shared information between the branches in different modalities. The fifth and sixth layers of the encoder extract high-level features that contain abundant global contextual information. Additionally, there is a significant similarity between the high-level features of both modalities. To fully exploit this common feature, we utilized a shared network to make the two branches have similar parameters in the last phase of the encoder, effectively decreasing the total number of parameters in the model. The shared network can exploit the similarities and synergies between different modes of data to correspond with the characteristics of the advanced cross-modal attributes.

#### 2.2.3. Cross-Modality Feature Aggregation Decoder

**Figure 6:**
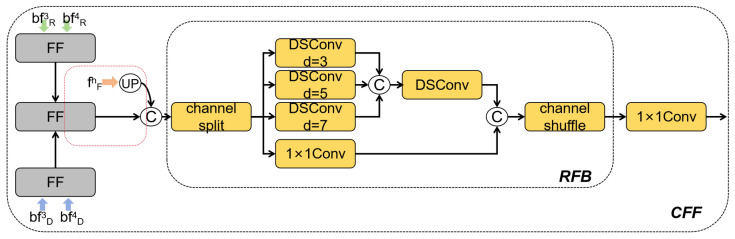
The complete architecture of the CFF.

As shown in Figure 4, the decoder consists of three cross-modal feature fusion (CFF) modules that make use of the output of the various layers of the encoder and combine their multi-scale features. Figure 6 illustrates the complete architecture of the CFF, comprising the FF module and the Receptive Field Block (RFB) (Liu and Huang, 2018) [21]. Using the CFF-middle module as an illustration, we initially combine the RGB features $bf_{R}^{i}\left(i=3,4\right)$ and depth features $bf_{D}^{i}\left(i=3,4\right)$ separately at multiple scales to acquire the subsequent features, as shown in Equations (4) and (5). Following the fusion of features at both levels, we proceed to perform cross-modality feature fusion to obtain further the advanced fused feature $f_{F}^{M}$ as illustrated in Equation (6).

(4) $$\begin{array}{c} \begin{array}{c} bf_{R}^{m}=FF\left(bf_{R}^{3},bf_{R}^{4}\right) \end{array} \end{array}$$

(5) $$\begin{array}{c} \begin{array}{c} bf_{D}^{m}=FF\left(bf_{D}^{3},bf_{D}^{4}\right) \end{array} \end{array}$$

(6) $$\begin{array}{c} \begin{array}{c} f_{F}^{m}=FF\left(bf_{R}^{m},bf_{D}^{m}\right) \end{array} \end{array}$$

After that, the fusion feature $f_{F}^{m}$ is concatenated with $f_{F}^{h}$ which is generated by the CFF-high module in the previous stage. The outcome of this operation is then fed into the RFB that utilizes channel split and shuffle operations within the receptive field. The features are partitioned into two parts along the channel dimension. One of them is fed into a 1×1 convolutional layer as the residual component, while the other part is input into a dilated convolution with multiple branches to extract multi-scale features. These two segments are then concatenated and subjected to a channel shuffle operation to facilitate information transfer between different channels. The resulting fused features, denoted as $f_{F}^{m}$ are obtained in the final CFF-middle layer.

The CFF module encompasses both the integration of cross-modality feature fusion and the fusion of multi-scale features from two modalities. This allows the model to fully utilize the shared characteristics and compatibility of the cross-modality features, enhancing the model’s ability to handle tasks that involve multiple scales and cross-modality interactions. Following the completion of three CFF modules, the resulting fused features are input into the regression module to obtain the estimated weight of the duck.

### 2.3. Training Details

In training, the network is trained for 100 epochs using the Pytorch framework (version 1.10.0, Paszke et al., 2019) [22], with an Intel(R) Core(TM) i9-9900K CPU @ 3.60 GHz and an RTX2080 Ti GPU with CUDA 11.5 architecture, and running Python 3.8.13. The RGB-D image pairs were uniformly resized to a dimension of 224 × 224. As part of the training process, we perform data augmentation, such as color dithering, random horizontal and vertical flipping, and random rotation. The optimizer used is AdamW (Loshchilov and Hutter, 2019) [23], the learning rate is set as 0.001, the learning rate weight decay is 0.05, and the batch size is 32.

### 2.4. Evaluation Metrics

We adopted *MAE*, *RMSE*, and $R^{2}$ as the metrics for evaluation purposes. The Mean Absolute Error (*MAE*) is commonly employed to assess the quality of regression outcomes, as it quantifies the precise discrepancy between the predicted and the actual values. *MAE* is computed using the following Formula (7), where $y_{i}$ denotes the actual value and ${\hat{y}}_{i}$ represents the estimated value.

(7) $$\begin{array}{c} \begin{array}{c} MAE=\frac{1}{n}\sum_{i=1}^{n}\left|{\hat{y}}_{i}-y_{i}\right| \end{array} \end{array}$$

Root Mean Square Error (*RMSE*) calculates the extent of discrepancy between the predicted value and the actual value, computing as Formula (8). A smaller *RMSE* value signifies a lower prediction error of the model and a higher predictive capability of the model.

(8) $$\begin{array}{c} \begin{array}{c} RMSE=\sqrt{\frac{1}{n}\sum_{i=1}^{n}{\left({\hat{y}}_{i}-y_{i}\right)}^{2}} \end{array} \end{array}$$

R-squared ($R^{2}$) is another statistical metric that evaluates how well the model fits the observed data, ranging between 0 and 1. A higher $R^{2}$ value indicates a stronger fit between the model and the data. The calculation of $R^{2}$ is as Formula (9), where ${\bar{y}}_{i}$ is the average true value of the test image.

(9) $$\begin{array}{c} \begin{array}{c} R^{2}=1-\frac{\sum_{i}{\left({\hat{y}}_{i}-y_{i}\right)}^{2}}{\sum_{i}{\left({\bar{y}}_{i}-y_{i}\right)}^{2}} \end{array} \end{array}$$

## 3. Results and Discussion

To evaluate the effectiveness of the proposed model, we conducted comparative experiments with single-modality and multi-modality methods as presented in Section 3.1 and Section 3.2. Besides, we analyzed the experimental results and researched the role of important modules of the proposed model in Section 3.4.

### 3.1. Comparison with Single-Modality Methods

By employing deep learning models on RGB or depth images, duck weight estimation can be accomplished with single-modality images. For a comparison study, we introduced several widely-used backbone networks, such as MobileNetV3, DenseNet (Iandola et al., 2014) [24], ResNet (He et al., 2016) [25], InceptionNet (Szegedy et al., 2016) [26], EfficientNet (Tan and Le, 2019) [27], and Swin Transformer (Liu et al., 2021) [28]. The outcomes of the comparison are presented in Table 1. The experimental results highlight the limitations of single-modality weight estimation methods, as all exhibit MAE exceeding 0.25. In contrast, the proposed multi-modality fusion method achieved a significantly lower MAE of 0.15, reducing errors by 62.9% compared to the RGB-only model and 78.7% versus the depth-only model. The RGB-based method is observed to outperform the depth-based method due to the richer semantic information of the RGB images. Besides, our proposed method consisting of both RGB and depth branches, which can complement each other’s features, demonstrates superior performance compared to the others. The MAE decreased by 0.2545 when compared to MobileNetV3 using a single RGB input and by 0.5536 when compared to MobileNetV3 using a single depth input.

### 3.2. Comparison with Multi-Modality Methods

**Figure 7:**
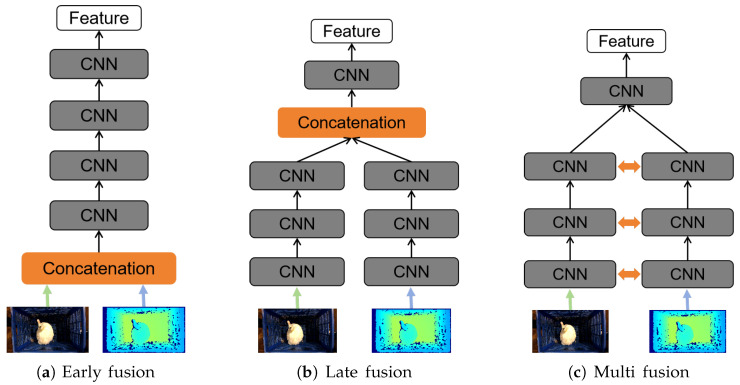
Comparison of three fusion strategies that explore the correlation between RGB images and depth images.

Currently, there are limited studies that utilize multi-modality images for the estimation of duck weight. Therefore, we selected three prominent networks that fuse multi-modality features in the field of RGB-D SOD for experimental comparison. The structures of these three feature extraction networks are illustrated in Figure 7. Figure 7a illustrates the early fusion process, in which the RGB and depth images are combined to create a four-channel image. This combined image is subsequently input into the feature extraction network to extract features. Figure 7b illustrates the later fusion process, where the output feature maps of the RGB and depth image are connected after being fed into the feature extraction network separately. Figure 7c illustrates the process of multi-scale fusion, which refers to the fusion strategy employed. Besides, we compared the work for pig weight estimation based on the two-stream network (He et al., 2023b) [13]. As indicated, our proposed algorithm outperforms the reproduced method in terms of accuracy and parameter amount. To evaluate these fusion means, we employ MobileNetv3, DenseNet, ResNet, and EfficientNet as the backbone. The results are presented in Table 2, indicating that the method proposed has superior performance in terms of *MAE*, *RMSE*, and $R^{2}$.

### 3.3. Visualizations and Analysis

It is essential to investigate whether the network is truly focusing on the region of interest in the duck image. Grad-CAM (Selvaraju et al., 2017) [29] can be used for determining the location of particular objects using a model that was trained only on image labels rather than explicit location annotations. We introduce it to visualize the salient CAM map of input duck images.

**Figure 8:**
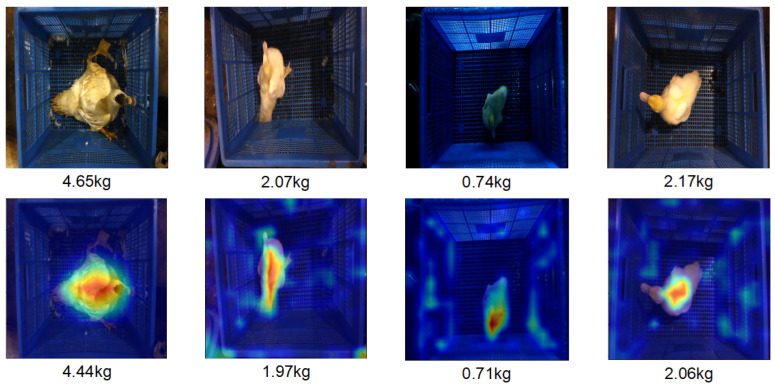
Row 1: Original RGB images and the ground-truth weight data; Row 2: Feature maps extracted by our algorithm and the estimated weight data.The warm-colored regions indicate the model’s focused attention zones.

As shown in Figure 8, the first row presents the original RGB images and real weight data, while the second row displays the feature maps extracted by our algorithm and the estimated weight data. The proposed method has achieved a relatively reasonable effect of duck recognition, and the attention is concentrated on the main area of the duck. This indicates the rationality and effectiveness of the proposed algorithm in the weight estimation task. The algorithm can summarize the living body features of ducks, thus providing an accurate and reasonable visual representation for weight estimation.

### 3.4. Discussion

#### 3.4.1. Result Analysis

In recent years, deep learning has demonstrated promising outcomes in the estimation of non-contact animal weight. The implementation of neural networks avoids capturing animals for weighing in a limited space. However, the majority of prior research has solely focused on one modality, ignoring the valuable supplementary data provided by other modalities. In contrast to the existing methods, our proposed approach utilizes both RGB images and depth images to learn cross-modality characteristics, enabling the better estimation of duck weight. The results from the comparison trials presented in Table 1 demonstrate that our method effectively decreases the MAE and RMSE and the value of $R^{2}$ achieves the highest value of 0.9682, in contrast to the single-modality methods. Furthermore, the utilization of neural networks eliminates the need for a pre-feature extraction step for the images.

Meanwhile, the proposed method utilizes a multi-scale fusion strategy to combine RGB-D cross-modality data. In this way, the branches of these two modalities interact with the other in both directions at each stage, which facilitates the mutual complementary of features from different modalities. Next, we use a decoder to combine the multi-scale features of multiple modalities to acquire more effective fusion features. The comparative results in Table 2 demonstrate that our method outperforms existing fusion processes under all metrics.

#### 3.4.2. Impact of CFS

In the feature extraction module, we incorporated the cross-modality feature supplementation (CFS) module to enable the two-stream network to acquire additional information from other modalities. To assess the effects of the CFS module, we evaluated the network’s performance without using the CFS. Meanwhile, we investigated the impact of adding different numbers of CFS on network performance. The experimental results in Table 3 demonstrate that our proposed approach achieves the best performance with improvements under several evaluation metrics, such as MAE, RMSE, and $R^{2}$. It is also observed that the model’s performance improves as the number of CFS increases from 1 to 4. However, the performance begins to decline when the fifth CFS is added, and it continues to decline as the number of CFS increases. This may be attributed to the similarity between the high-level features extracted from the fifth and sixth layers of the model from the two modalities. Consequently, there is no need to augment the information from the other modalities. This demonstrates the rationality of establishing four CFS modules and designating layers 5 and 6 as a shared network.

#### 3.4.3. Impact of CFF

We employ a cross-modality feature fusion module with the decoder structure to combine features, rather than some commonly used strategies for feature fusion, e.g., addition, maxing, or concatenation operations. The comparative results are displayed in Table 4, revealing that the proposed method outperforms the other three fusion schemes. The result shows that by merging multi-modality features of multiple sizes, the model could effectively blend the retrieved features from both modalities and provide more precise fused features.

#### 3.4.4. Limitations

The above experimental results indicate that the proposed method can effectively utilize RGB and depth features for duck weight estimation, but it inevitably has some shortcomings. First of all, because the dataset was created by shooting from a top-down perspective, the duck poses were limited to lying down and standing; other poses, like ducks spreading their wings, were not included. This study did not examine the influence factor regarding the duck poses, and dynamic gestures were not covered due to the limitation of the dataset, so it will be important to consider it in future work. In addition, the second limitation pertains to the quality of the acquired depth data. Despite our efforts to optimize these data through various pre-processing steps, the resulting depth images inevitably encounter some quality issues due to the inherent limitations of the experimental equipment, fluctuations in the surrounding environment (e.g., uneven illumination) and uncontrollable factors during data acquisition, etc. These issues, specifically blurry edges, distorted depth information and inadequate resolution, can significantly compromise the precision and dependability of subsequent assessments. Future research will thus explore more robust data processing methodologies and efficient data acquisition techniques, aiming to elevate the quality of depth map data and foster continued progress in related fields.

## 4. Conclusions

Our proposal involves the employment of RGB-D images to create a cross-modality bi-directional interaction network that benefits cross-modality feature complementation. The MobileNetv3 serves as the backbone for the two branches. In the feature extraction module, a cross-modality feature supplementation module is employed to facilitate learning between the two modalities. Besides, a cross-modality feature fusion module is designed to combine the multi-scale features extracted from the two branches as the decoder. Finally, the fused features are input into a regression module to estimate the weight of the duck. For our research, we established a novel dataset of RGB-D duck images, named SCAU-DuckWT, to estimate duck weight. We performed comparative and ablation experiments on the dataset to assess the effectiveness of our proposed approach. The results demonstrate that our method exhibits superior performance, with an MAE of 0.1550.

In future work, we will update the data acquisition and processing means to improve the quality of RGB-D images aiding in training the model. Besides, we will explore more accurate weight estimation methods to cope with a wider variety of duck poses. In subsequent work, we will focus on dynamic behaviors and analyze the impact of these behaviors on the results of the weight estimation model, and we will take the actual farm environment into account. We plan to conduct experiments in the farm environment to explore the feasibility of practical applications. Additionally, the non-contact weight measurement methods based on deep learning would be generalized to other poultry and beneficial for automatic monitoring in the poultry farming industry. Our model is founded on RGB-D image fusion and cross-modal feature learning, which are general technical means. For other poultry, such as chickens and geese, we can also obtain rich information through RGB-D images, and thus our model can be used for a broader range of weight estimation scenarios.

## Figures and Tables

**Table 1:** The comparative results with single-modality methods on the RGB-D dataset.

Method	Modality	FLOPS	Parameters	MAE	RMSE	$R^{2}$
MobileNetV3	RGB	16.26 G	1.13 M	0.4095	0.6525	0.7323
DenseNet	RGB	87.59 G	1.01 M	0.2540	0.3848	0.9069
ResNet	RGB	55.20 G	21.69 M	0.3403	0.5152	0.8331
InceptionNet	RGB	354.51 G	6.88 M	0.2971	0.4161	0.8911
EfficientNet	RGB	5.35 G	2.49 M	0.2723	0.3706	0.9136
Swin Transformer	RGB	65.43 G	28.27 M	0.3887	0.6021	0.7721
MobileNetV3	Depth	16.20 G	1.13 M	0.7086	0.8899	0.5021
DenseNet	Depth	85.86 G	1.01 M	0.7138	0.8779	0.5154
ResNet	Depth	54.02 G	21.69 M	0.6164	0.7897	0.6078
InceptionNet	Depth	354.42 G	6.88 M	0.9377	1.0525	0.3034
EfficientNet	Depth	5.24 G	2.49 M	0.7542	0.9079	0.4817
Swin Transformer	Depth	65.28 G	28.27 M	0.7627	0.9323	0.4535
Proposed Method	RGB + Depth	37.13 G	6.83 M	0.1550	0.2250	0.9682

**Table 2:** The comparative results with multi-modality methods on the RGB-D dataset.

Method	FLOPS	Parameters	MAE	RMSE	$R^{2}$
MobileNetV3 (Early)	16.37 G	1.13 M	0.3099	0.4668	0.863
DenseNet (Early)	88.42 G	1.01 M	0.3015	0.4543	0.8702
ResNet (Early)	55.70 G	21.96 M	0.3294	0.4686	0.8619
EfficientNet (Early)	5.39 G	2.49 M	0.2971	0.3886	0.9051
MobileNetV3 (Late)	32.48 G	red2.26 M	0.3203	0.4380	0.8794
DenseNet (Late)	173.435 G	2.03 M	0.2778	0.4190	0.8896
ResNet (Late)	109.209 G	red43.90 M	0.3096	0.4111	0.8937
EfficientNet (Late)	10.575 G	5.01 M	0.3341	0.4745	0.8584
Pig weight net	8.70 G	55.0 M	0.1550	0.2250	0.9682
(multi fusion)					
Proposed Method	16.26 G	1.13 M	0.1550	0.2250	0.9682

**Table 3:** The results of different number of CFS, where CFS denotes cross-modality feature supplement.

Method	MAE	RMSE	$R^{2}$
two-stream MobileNetv3 w/o CFS	0.1928	0.2581	0.9581
two-stream MobileNetv3 w 1CFS	0.1931	0.2685	0.9547
two-stream MobileNetv3 w 2CFS	0.1751	0.2355	0.9651
two-stream MobileNetv3 w 3CFS	0.1622	0.2347	0.9654
two-stream MobileNetv3 w 5CFS	0.1949	0.2703	0.9541
two-stream MobileNetv3 w 6CFS	0.1828	0.2500	0.9607
Proposed Method	0.1550	0.2250	0.9682

**Table 4:** The result of three fusion schemes.

Method	MAE	RMSE	$R^{2}$
Addition	0.1952	0.272	0.9601
Max	0.2156	0.2948	0.9453
Concatenation	0.2126	0.2909	0.9468
Proposed Method	0.1550	0.2250	0.9682

## Data Availability

The original contributions presented in this study are included in the article. Further inquiries can be directed to the corresponding author(s).

## References

[1] Jalaludeen A., Churchil R.R., Jalaludeen A., Churchil R.R., Baéza E. (2022). Duck Production: An Overview. Duck Production and Management Strategies.

[2] Téguia A., Ngandjou H.M., Defang H., Tchoumboue J. (2008). Study of the live body weight and body characteristics of the African Muscovy duck (*Caraina moschata*). Trop. Anim. Health Prod..

[3] Li G., Zhao Y., Purswell J.L., Du Q., Chesser G.D., Lowe J.W. (2020). Analysis of feeding and drinking behaviors of group-reared broilers via image processing. Comput. Electron. Agric..

[4] Xiao D., Wang H., Liu Y., Li W., Li H. (2024). DHSW-YOLO: A duck flock daily behavior recognition model adaptable to bright and dark conditions. Comput. Electron. Agric..

[5] Ogah D.M., Yakubu A., Momoh M.O., Dim N.I. (2011). Relationship between some body measurements and live weight in adult Muscovy ducks using path analysis. Trakia J. Sci..

[6] Lieng P., Sangpradit K. (2020). Study on duck weight estimation by using image processing. Proceedings of the E3S Web of Conferences.

[7] Chen R., Zhao Y., Yang Y., Wang S., Li L., Sha X., Liu L., Zhang G., Li W.J. (2023). Online estimating weight of white Pekin duck carcass by computer vision. Poult. Sci..

[8] Pezzuolo A., Guarino M., Sartori L., González L.A., Marinello F. (2018). On-barn pig weight estimation based on body measurements by a Kinect v1 depth camera. Comput. Electron. Agric..

[9] Cang Y., He H., Qiao Y. (2019). An intelligent pig weights estimate method based on deep learning in sow stall environments. IEEE Access.

[10] He H., Qiao Y., Li X., Chen C., Zhang X. (2021). Automatic weight measurement of pigs based on 3D images and regression network. Comput. Electron. Agric..

[11] Antonakakis M., April T., Bailey M., Bernhard M., Bursztein E., Cochran J., Durumeric Z., Halderman J.A., Invernizzi L., Kallitsis M. Understanding the mirai botnet. Proceedings of the 26th USENIX Security Symposium (USENIX Security 17).

[12] He C., Qiao Y., Mao R., Li M., Wang M. (2023). Enhanced LiteHRNet based sheep weight estimation using RGB-D images. Comput. Electron. Agric..

[13] He W., Mi Y., Ding X., Liu G., Li T. (2023). Two-stream cross-attention vision Transformer based on RGB-D images for pig weight estimation. Comput. Electron. Agric..

[14] Zhou T., Fan D.P., Cheng M.M., Shen J., Shao L. (2021). RGB-D salient object detection: A survey. Comput. Vis. Media.

[15] Qu L., He S., Zhang J., Tian J., Tang Y., Yang Q. (2017). RGBD salient object detection via deep fusion. IEEE Trans. Image Process..

[16] Han J., Chen H., Liu N., Yan C., Li X. (2017). CNNs-based RGB-D saliency detection via cross-view transfer and multiview fusion. IEEE Trans. Cybern..

[17] Chen H., Li Y., Su D. (2019). Multi-modal fusion network with multi-scale multi-path and cross-modal interactions for RGB-D salient object detection. Pattern Recognit..

[18] Wang Y., Zhang Y. Three-stage bidirectional interaction network for efficient RGB-D salient object detection. Proceedings of the Asian Conference on Computer Vision.

[19] Howard A., Sandler M., Chu G., Chen L.C., Chen B., Tan M., Wang W., Zhu Y., Pang R., Vasudevan V. Searching for mobilenetv3. Proceedings of the IEEE/CVF International Conference on Computer Vision.

[20] Park J., Woo S., Lee J.Y., Kweon I.S. (2018). BAM: Bottleneck Attention Module. arXiv.

[21] Liu S., Huang D. Receptive field block net for accurate and fast object detection. Proceedings of the European Conference on Computer Vision (ECCV).

[22] Paszke A., Gross S., Massa F., Lerer A., Bradbury J., Chanan G., Killeen T., Lin Z., Gimelshein N., Antiga L. Pytorch: An imperative style, high-performance deep learning library. Proceedings of the Advances in Neural Information Processing Systems 32 (NeurIPS 2019).

[23] Loshchilov I., Hutter F. (2019). Decoupled Weight Decay Regularization. arXiv.

[24] Iandola F., Moskewicz M., Karayev S., Girshick R., Darrell T., Keutzer K. (2014). DenseNet: Implementing Efficient ConvNet Descriptor Pyramids. arXiv.

[25] He K., Zhang X., Ren S., Sun J. Deep residual learning for image recognition. Proceedings of the IEEE Conference on Computer Vision and Pattern Recognition.

[26] Szegedy C., Vanhoucke V., Ioffe S., Shlens J., Wojna Z. Rethinking the inception architecture for computer vision. Proceedings of the IEEE Conference on Computer Vision and Pattern Recognition.

[27] Tan M., Le Q. (2019). Efficientnet: Rethinking model scaling for convolutional neural networks. Proceedings of the International Conference on Machine Learning.

[28] Liu Z., Lin Y., Cao Y., Hu H., Wei Y., Zhang Z., Lin S., Guo B. Swin transformer: Hierarchical vision transformer using shifted windows. Proceedings of the IEEE/CVF International Conference on Computer Vision.

[29] Selvaraju R.R., Cogswell M., Das A., Vedantam R., Parikh D., Batra D. Grad-cam: Visual explanations from deep networks via gradient-based localization. Proceedings of the IEEE International Conference on Computer Vision.

